# Evaluating the distinct pleiotropic effects of omega-3 fatty acids on type 2 diabetes mellitus: a mendelian randomization study

**DOI:** 10.1186/s12967-023-04202-7

**Published:** 2023-06-07

**Authors:** Chunyan Hu, Yulin Zhou, Xueyan Wu, Xiaojing Jia, Yuanyue Zhu, Ruizhi Zheng, Shuangyuan Wang, Lin Lin, Hongyan Qi, Hong Lin, Mian Li, Tiange Wang, Zhiyun Zhao, Min Xu, Yu Xu, Yuhong Chen, Guang Ning, Maria-Carolina Borges, Weiqing Wang, Jie Zheng, Yufang Bi, Jieli Lu

**Affiliations:** 1 Department of Endocrine and Metabolic Diseases, Shanghai Institute of Endocrine and Metabolic Diseases, Ruijin Hospital, Shanghai Jiao Tong University School of Medicine, 197 Rui Jin 2nd Road, Shanghai, 200025 China; 2 Shanghai National Clinical Research Center for Metabolic Diseases, Key Laboratory for Endocrine and Metabolic Diseases of the National Health Commission of the PR China, Shanghai Key Laboratory for Endocrine Tumor, State Key Laboratory of Medical Genomics, Ruijin Hospital, Shanghai Jiao Tong University School of Medicine, Shanghai, China; 3 MRC Integrative Epidemiology Unit (IEU), Bristol Medical School, University of Bristol, Oakfield House, Oakfield Grove, Bristol, BS8 2BN UK; 4 Population Health Sciences, Bristol Medical School, University of Bristol, Bristol, UK

**Keywords:** Omega-3 fatty acids, Type 2 diabetes, Mendelian randomization, Pleiotropic effects, Genetic epidemiology, Glucose metabolism

## Abstract

**Background:**

Observational studies and conventional Mendelian randomization (MR) studies showed inconclusive evidence to support the association between omega-3 fatty acids and type 2 diabetes. We aim to evaluate the causal effect of omega-3 fatty acids on type 2 diabetes mellitus (T2DM), and the distinct intermediate phenotypes linking the two.

**Methods:**

Two-sample MR was performed using genetic instruments derived from a recent genome-wide association study (GWAS) of omega-3 fatty acids (N = 114,999) from UK Biobank and outcome data obtained from a large-scale T2DM GWAS (62,892 cases and 596,424 controls) in European ancestry. MR-Clust was applied to determine clustered genetic instruments of omega-3 fatty acids that influences T2DM. Two-step MR analysis was used to identify potential intermediate phenotypes (e.g. glycemic traits) that linking omega-3 fatty acids with T2DM.

**Results:**

Univariate MR showed heterogenous effect of omega-3 fatty acids on T2DM. At least two pleiotropic effects between omega-3 fatty acids and T2DM were identified using MR-Clust. For cluster 1 with seven instruments, increasing omega-3 fatty acids reduced T2DM risk (OR: 0.52, 95%CI 0.45–0.59), and decreased HOMA-IR (β = − 0.13, SE = 0.05, P = 0.02). On the contrary, MR analysis using 10 instruments in cluster 2 showed that increasing omega-3 fatty acids increased T2DM risk (OR:1.10; 95%CI 1.06–1.15), and decreased HOMA-B (β = − 0.04, SE = 0.01, P = 4.52 × 10^–5^). Two-step MR indicated that increasing omega-3 fatty acid levels decreased T2DM risk via decreasing HOMA-IR in cluster 1, while increased T2DM risk via decreasing HOMA-B in cluster 2.

**Conclusions:**

This study provides evidence to support two distinct pleiotropic effects of omega-3 fatty acids on T2DM risk influenced by different gene clusters, which could be partially explained by distinct effects of omega-3 fatty acids on insulin resistance and beta cell dysfunction. The pleiotropic feature of omega-3 fatty acids variants and its complex relationships with T2DM need to be carefully considered in future genetic and clinical studies.

**Supplementary Information:**

The online version contains supplementary material available at 10.1186/s12967-023-04202-7.

## Introduction

Over the past decades, diabetes has become a prevalent public health challenge [1]. 537 million adults had been affected in 2021 and approximately 783 million adults were predicted to have diabetes in 2045 [2]. Type 2 diabetes mellitus (T2DM) is an important risk factor for cardiovascular disease and mortality [3]. Glucose level, as a biomarker for T2DM, is normally regulated by a feedback loop including islet beta cells and insulin-sensitive tissues, while insulin resistance and beta cell dysfunction are two major pathological features of T2DM [4]. As a complex disease, the causal risk factors of T2DM are still not fully understood. Among its intricate triggers, diet is a critical cornerstone in the prevention, delay, and management of T2DM [5].

Omega-3 fatty acids, commonly found in seafood or some plant oils, was previously acknowledged as a dietary supplement for its potential favorable effects on cardiovascular risk [6]. However, some studies have showed conflict results, suggesting that omega-3 fatty acids have no benefit in the prevention of cardiovascular disease, or its protective effect is negligible [7, 8]. Moreover, existing clinical trials and observational studies provided heterogenous evidence to support the role of omega-3 fatty acids on T2DM [9–14]. A recent large-scale prospective study indicated that circulating omega-3 fatty acids was associated with lower risk of T2DM [10]. On the contrary, some studies suggested little evidence to support its prevention role on T2DM and glucose metabolism [9], or the benefits were only efficient in certain populations [11] or on specific sources of omega-3 fatty acids [12]. Some studies even suggested an increased risk of T2DM [13, 14]. Previous studies further indicated that omega-3 fatty acids could preserve insulin sensitivity, lower triglycerides, and reduce inflammatory mediators [15–17], which implies that omega-3 fatty acids might influence T2DM through these pathways.

Large-scale RCT is the gold standard approach to estimate the causality of omega-3 fatty acids on T2DM. However, it is expensive and time-consuming, which restricted its application. Mendelian randomization (MR) uses genetic variants as instruments to estimate the causal effect of an exposure on an outcome. MR is generally not susceptible to confounding or reverse causation since alleles of genes are randomly assigned from parents to offspring during meiotic process, according to the Mendel’s law of independent assortment [18]. MR has previously been used to evaluate the causal effect of fatty acids on non-communicable chronic diseases risk [19–22]. Till now, one MR study suggested null effect of omega-3 fatty acids on T2DM [23], while another study focused on the individual components of omega-3 fatty acids and suggested that some increased while some others decreased T2DM risk [24]. Previous genetic studies have showed that some omega-3 fatty acids associated genes, such as *FADS* genes, was pleiotropically associated with multiple fatty acids [20, 22], while other omega-3 fatty acids associated genes such as *GCKR* are well-known pleiotropic genes that associated with multiple diseases, including T2DM [24]. Most of these MR studies have not systematically investigated the pleiotropic mechanisms of omega-3 fatty acids, which could bias the MR estimates. MR-clust is a novel method that identify clusters of variants with heterogeneous effects to reflect different causal pathways [25]. MR-clust has the potential to provide new biological insights for T2DM aetiology and improve our understanding on how pleiotropic features of omega-3 fatty acids instruments may influence its causal estimate on T2DM.

The aim of the present study was to examine the causal effect of circulating omega-3 fatty acids on risk of T2DM, establish potential distinct causal mechanisms of omega-3 fatty acids on T2DM and interpret the heterogeneity/pleiotropy pattern of omega-3 and T2DM.

## Methods

### Study design

**Fig. 1:**
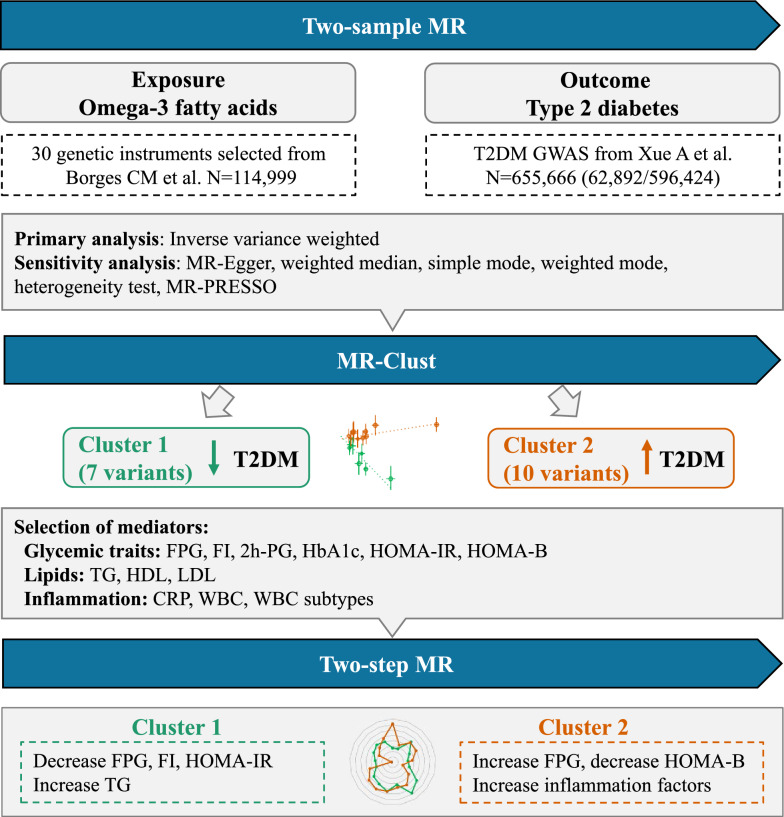
Study Design. *FPG* fasting plasma glucose, *FI* fasting insulin, *2 h-PG* 2 h-postload glucose; *TG* triglycerides, *CRP* C-reactive protein, *WBC* white blood cell, *MR* Mendelian Randomization, *T2DM* type 2 diabetes

Figure 1 illustrates the overview of this study. First, a two-sample MR analysis was conducted to estimate the overall effect of omega-3 fatty acids on T2DM using publicly available genome wide association studies (GWAS; Additional file 1: Table S1). Ethics committee approval and participant informed consent was obtained in the original studies. Second, MR-Clust was used to assess the distinct causal effects by which omega-3 fatty acids may influence T2DM [25]. Third, a two-step MR was conducted to identify potential intermediate phenotypes linking omega-3 fatty acids with T2DM in different clusters. Glycemic traits, lipid profiles and inflammatory indicators were selected as the intermediate phenotypes for the two-step MR since omega-3 fatty acids were reported to be associated with these phenotypes [15–17]. This study is reported as per the Strengthening the Reporting of Observational Studies in Epidemiology (STROBE) guideline, specific for MR.

### Instrument selection for omega-3 fatty acids

SNPs associated with total omega-3 fatty acids were obtained from a large-scale UK Biobank GWAS [21] of fatty acids with 114,999 individuals of European ancestry. The mean concentration of total omega-3 fatty acids was 0.53 (SD 0.22) mmol/L. After excluding missing SNPs in the outcome data, 30 SNPs showed robust association with omega-3 fatty acids (with genetic association P value < 5 × 10^–8^) and showed independence (with linkage disequilibrium [LD] r^2^ < 0.001), which were selected as instruments for the MR analyses. Detailed information of the genetic instruments was present in the Additional file 1: Table S2.

### Outcome selection

For each omega-3 fatty acids instrument, the genetic effect of the corresponding SNP on T2DM was obtained from the most recent GWAS with a total of 655,666 Europeans (62,892 T2DM cases and 596,424 controls) [26]. Among them, 21,147 (33.6%) T2DM cases were from UK Biobank.

### Selection of intermediate phenotypes

We searched PubMed and Embase from inception up to Oct 10, 2022 using the search terms: “fish oil”, “omega-3”, or “omega 3 fatty acid”, without language restrictions. Lipid profiles and inflammation factors were included as intermediate phenotypes after the search [15–17]. In addition, glycemic phenotypes were included since their strong links with our primary outcome T2DM. Therefore, six glycemic traits [fasting plasma glucose (FPG), fasting insulin (FI), 2 h-postload glucose (2 h-PG), HbA1c, HOMA-IR and HOMA-B], three lipid traits [triglycerides, LDL and HDL], and seven inflammatory indicators [C-reactive protein (CRP), white blood cell (WBC) and five WBC subtypes] were considered as potential intermediate phenotypes that may link omega-3 fatty acids with T2MD (Additional file 1: Table S1).

### Statistical analyses

#### MR analysis

The summary genetic associations data sets were harmonized using the “*TwosampleMR*” [27] package. The inverse variance weighted (IVW) [28] method was used to estimate the genetically predicted effect of omega-3 fatty acids on T2DM in the primary MR analyses. The ORs and 95% CIs of T2DM with per unit increasement in omega-3 fatty acids were calculated.

#### MR-Clust analysis

Next, MR-Clust was used to dissect heterogeneity in the estimated causal effects of omega-3 fatty acids on T2DM [25]. This algorithm accounts for differential uncertainty in the causal estimates, and divided all variants with similar causal estimates into distinct clusters. 30 genetic variants based on their causal estimates for omega-3 fatty acids and T2DM were used for the clustering processes. Cluster with at least four variants with the conditional probability ≥ 0.8 assigned to the cluster were selected. After clustering, two substantial clusters were identified. The MR effect estimates and the levels of heterogeneity and pleiotropy were evaluated in each cluster separately.

#### Two-step MR analysis

The two-step MR was applied to identify potential intermediate phenotypes linking omega-3 fatty acids with T2DM in each of the two identified clusters, which could indicate pleiotropic pathways and/or distinct biological mechanisms in each cluster. The above-mentioned 16 intermediate phenotypes were tested in this analysis. In the first step of the two-step MR, we used the IVW method to investigate the causal effect of omega-3 fatty acids on the intermediate phenotype as estimated by the SNPs belonging to that cluster. For MR estimates that showed strong evidence of heterogeneity, we used Radial MR [29] to identify and remove outliers to reduce heterogeneity. Intermediate phenotypes showing MR evidence (IVW P value < 0.05) in each cluster in the first step were selected as candidates for the second step MR. In the second step, we used multivariable MR models to estimate the direct effects of omega-3 fatty acids and intermediate phenotypes selected in step one on the risk of T2DM. These analyses included SNPs selected for omega-3 fatty acids and for each of the intermediate phenotypes (with LD r^2^ < 0.001 and P < 5 × 10^–8^). Given limited power of the relevant GWAS, a more lenient threshold of P < 1 × 10^–6^ was used to identify instruments for HOMA-IR.

#### Assessment of MR assumptions

MR analysis assumes that a genetic variant used proxy traits 1) is robustly associated with the exposure (“relevance”); 2) is not associated with confounders of the instrument-outcome relationship (“exchangeability”); 3) has no effect on the outcome except through the exposure (“exclusion restriction”). To test these core MR assumptions, we performed the following sensitivity analysis.

The relevance assumption for MR was tested by generating estimates of the proportion of variance for the omega-3 fatty acids explained by the instrument (R^2^) and F statistics. Conditional F statistic within the multivariable MR analysis was used to quantify instrument strength. An F-statistic value of at least 10 could indicate evidence to against weak instrument bias.

To test the MR assumption of exclusion restriction, we evaluated the horizontal pleiotropy for the association between omega-3 fatty acids and T2DM. The presence of horizontal pleiotropy, which means a genetic variant influences an unintended phenotype through biological pathways that are independent of the exposure, is a violation of the exclusion restriction criterion. Therefore, sensitivity analyses, including MR-Egger [30], weighted median [31], simple mode, and weighted mode [32] method were performed to validate the IVW results. MR-Egger intercept and MR Pleiotropy RESidual Sum and Outlier test (MR-PRESSO) [33] were used to estimate levels of horizontal pleiotropy. Presence of heterogeneity was assessed using Cochrane’s Q test for IVW analyses and Rücker’s Q test for MR-Egger analyses [34, 35]. In addition, we used MR-Clust to characterize and better understand the potential pleiotropic nature of omega-3 fatty acids genetic variants.

All statistical tests were 2-sided and the significance threshold was set at P < 0.05. We also report the associations with p-values < 0.05 after Benjamini–Hochberg false discovery rate (FDR) correction in the two-step MR. All statistical analyses were performed using R (version 4.1.2). All the MR analyses were conducted using the “*TwosampleMR* (version 0.5.6)” [27], “*mrclust*” [25], “*RadialMR*” [29], “*MVMR*” [36] R package.

## Results

Genetic instruments for omega-3 fatty acids used in this study were listed in Additional file 1: Table S2. F statistics ranged from 26.16 to 6315.26, suggesting that the estimates were not likely subject to weak instrument bias. Instruments used in the multivariable MR analysis were presented in Additional file 2: Table S3. The conditional F statistics within the multivariable MR analyses indicated that there was little probability of weak instrumental bias, except for HOMA-IR with the conditional F statistic of 6.71. We kept HOMA-IR in our analysis but with the understanding that the genetic predictors could be influenced by weak instrument bias.

### Effect of omega-3 fatty acids on T2DM

The MR estimates using IVW (OR: 0.97; 95%CI 0.87–1.08; P = 0.60) and simple mode method (OR: 0.78; 95%CI 0.57–1.06; P = 0.12) showed a tendency of negative association between omega-3 fatty acids and T2DM, while the weighted median and weighted mode approaches showed that increasing levels of omega-3 fatty acids increased risk of T2DM (OR: 1.08; 95% CI 1.03–1.13, P = 0.002) (Table 1 and Additional file 1: Fig S1). There was substantial imprecision in MR estimates and 95% CI overlapped between different methods. Strong heterogeneity across the genetic instruments was observed using both Cochrane’s Q test (P = 4.16 × 10^–37^) and Rücker’s Q test (P = 2.71 × 10^–34^; Table 1), suggesting robust evidence to support heterogenous effects of omega-3 fatty acids on T2DM risk. MR-PRESSO also provided evidence for the existence of overall horizontal pleiotropy among instruments of omega-3 fatty acids (MR-PRESSO global test P < 0.001).

**Table 1:** Mendelian randomization results of causal effect of omega-3 fatty acids on type 2 diabetes in different clusters

	Method	No. SNPs	Causal estimate		Heterogeneity		Pleiotropy	
			OR (95% CI)	*P*	Q	*P*	Intercept	*P*
Total	IVW	30	0.97 (0.87–1.08)	0.600	250.45	4.16 × 10^–37^	–	–
	MR Egger	30	1.05 (0.90–1.21)	0.556	233.47	2.71 × 10^–34^	− 0.0095	0.165
	Weighted median	30	1.08 (1.03–1.13)	0.002	–	–	–	–
	Simple mode	30	0.78 (0.57–1.06)	0.120	–	–	–	–
	Weighted mode	30	1.08 (1.03- 1.13)	0.002	–	–	–	–
Cluster 1	IVW	7	0.52 (0.45–0.59)	2.91 × 10^–21^	10.33	0.111	-	-
	MR Egger	7	0.55 (0.43–0.71)	0.006	9.57	0.088	-0.0055	0.555
	Weighted median	7	0.59 (0.50–0.69)	6.62 × 10^–11^	–	–	–	–
	Simple mode	7	0.60 (0.43–0.83)	0.023	–	–	–	–
	Weighted mode	7	0.60 (0.49–0.73)	0.002	–	–	–	–
Cluster 2	IVW	10	1.10 (1.06–1.15)	4.41 × 10^–6^	6.13	0.727	–	–
	MR Egger	10	1.09 (1.03–1.16)	0.021	5.73	0.677	0.0025	0.549
	Weighted median	10	1.10 (1.05–1.15)	0.0001	–	–	–	–
	Simple mode	10	1.04 (0.91–1.19)	0.560	–	–	–	–
	Weighted mode	10	1.09 (1.04–1.15)	0.006	–	–	–	–

*IVW* inverse variance weighted, *OR* odds ratio, *CI* confidence interval

### Clustering effect of omega-3 fatty acids on T2DM

**Fig. 2:**
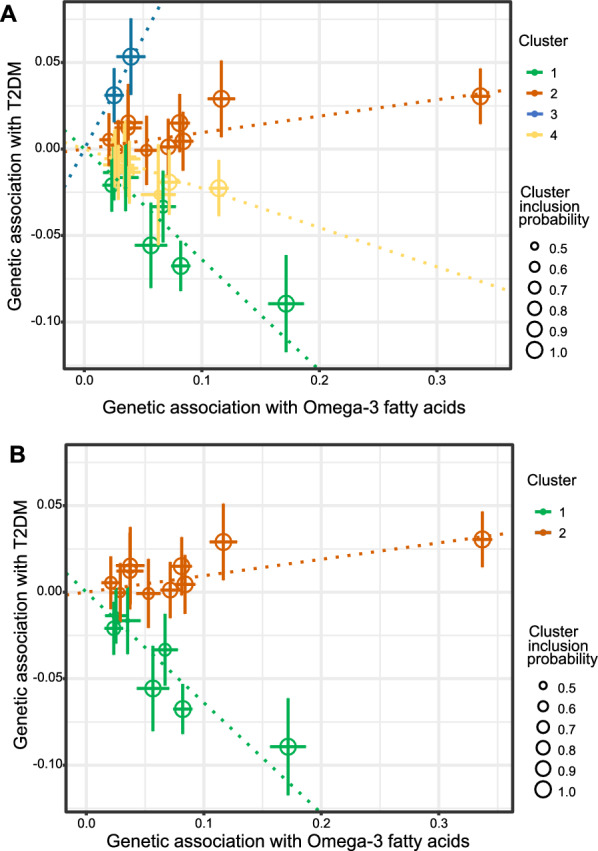
Clustered genetic associations with omega-3 fatty acids and type 2 diabetes. Each genetic variant is represented by a point. **A**. Each variant is assigned to the cluster with the greatest conditional probability. **B**. Only clusters having at least 4 variants with the conditional probability ≥ 0.8. T2DM, type 2 diabetes.

Based on the MR-Clust method, the 30 genetic instruments associated with omega-3 fatty acids were classified into four clusters (Fig. 2A). After filtered by variant number and conditional probability over 0.8, two distinct clusters remained, in which cluster 1 showed a negative effect and cluster 2 showed a positive effect on T2DM (Fig. 2B). Details of the genetic variants and assignment to clusters are shown in Additional file 1: Table S2. Cluster 1 with 7 variants suggested that omega-3 fatty acids have a negative effect on T2DM risk (OR 0.52; 95%CI 0.45–0.59, P = 2.91 × 10^–21^). On the contrary, cluster 2 comprised 10 instruments showed a positive effect (OR 1.10; 95%CI 1.06–1.15; P = 4.41 × 10^–6^). After applying the cluster approach, the observed heterogeneity in the main analysis was massively attenuated (P value of Cochrane’s Q statistics = 0.11; P value of Rücker’s Q statistics = 0.09). Little evidence was observed to support directional pleiotropy (P_MR-Egger intercept_ = 0.555; Table 1).

### Exploring distinct biochemical mechanisms and/or pleiotropic pathways of omega-3 fatty acids on T2DM

**Fig. 3:**
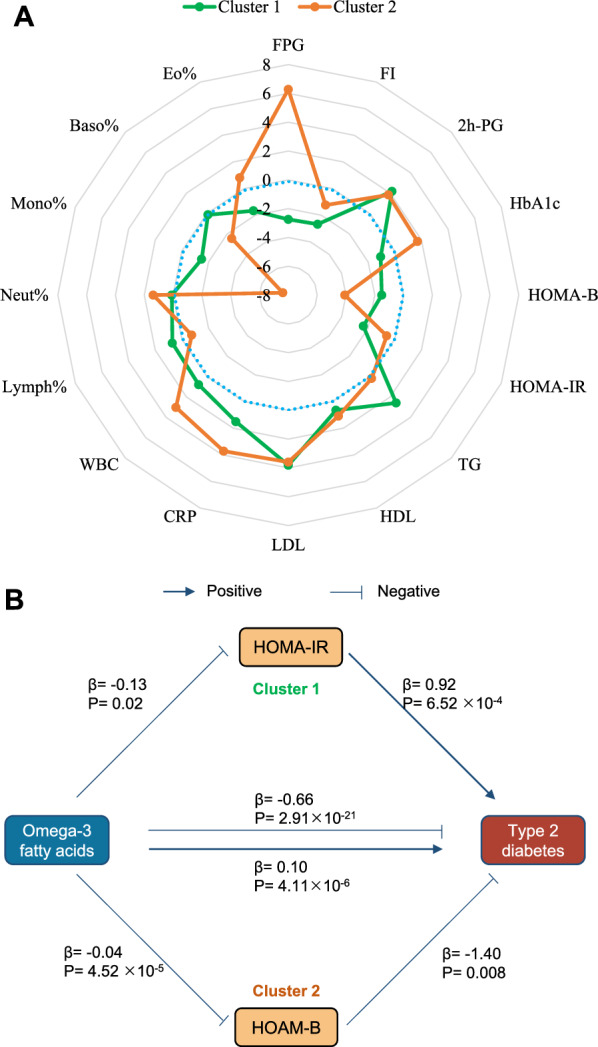
Two-step MR analysis identified potential intermediate phenotypes that linking omega-3 fatty acids with type 2 diabetes and potential pleiotropic pathways and/or distinct biological mechanisms in each cluster. A. Causal association of omega-3 fatty acids on potential mediators in different clusters. Estimated was presented as Z-score. *FPG* fasting plasma glucose, *FI* fasting insulin; 2 h-PG, 2 h-postload glucose, *TG* triglycerides, *CRP*, C-reactive protein, *WBC* white blood cell. Baso%, Basophil percentage; Eo%, Eosinophil percentage; Lymph%, Lymphocyte percentage; Mono%, Monocyte percentage; Neut%, Neutrophil percentage. **B**. Summary of the effect of insulin resistance (HOMA-IR) and beta cell dysfunction (HOMA-B) on the distinct pathways for omega-3 fatty acids on type 2 diabetes risks in the two clusters.

A two-step MR was applied to explore potential distinct biochemical mechanisms and/or pleiotropic pathways between omega-3 fatty acids and T2DM. In the first step, casual effects of omega-3 fatty acids in each cluster on intermediate phenotypes, including glycemic traits, lipid profiles and inflammation indicators, were estimated (Additional file 1: Table S4). Figure 3A intuitively displayed distinct causal effects of omega-3 fatty acids on the tested intermediate phenotypes in the two distinct clusters. For glycemic traits, at an FDR-corrected p-value < 0.05, we observed that increasing omega-3 fatty acid levels showed an effect on decreased FPG levels (β = -0.18; SE = 0.07; P = 0.006), FI levels (β = − 0.16; SE = 0.06; P = 0.007) and HOMA-IR levels (β = − 0.13; SE = 0.05; P = 0.018) in cluster 1. In contrast, increasing omega-3 fatty acid concentrations showed an effect on increased FPG levels (β = 0.04; SE = 0.07; P = 3.49 × 10^–10^) and decreased HOMA-B levels (β = − 0.04; SE = 0.01; P = 4.52 × 10^–5^) in cluster 2. For lipid profiles, increased omega-3 fatty acids showed an effect on increased triglycerides levels (β = 0.67; SE = 0.26; P = 0.01) in cluster 1. Since large heterogeneity (P value of Q statistics < 0.05, MR-PRESSO global test P < 0.001) and over-dispersion were detected in cluster 2 (Additional file 1: Fig S2), we removed two variants in the IVW analysis and the results after removal suggested that omega-3 fatty acids marginally decreased triglycerides levels in cluster 2 (β = -0.12; SE = 0.06; P = 0.03, FDR = 0.05). Besides, increasing omega-3 fatty acids showed an effect on increased LDL levels in both cluster 1 and cluster 2. Distinguished effect on inflammatory biomarkers across two cluster was also detected. Modest increased effect on CRP (β = 0.05; SE = 0.01; P = 1.95 × 10^–4^) and WBC (β = 0.05; SE = 0.02; P = 0.002) was observed in cluster 2. In summary, increasing omega-3 fatty acids (and related instruments) in cluster 1 shown a protective effect on glycemic traits, but a harmful effect on lipids, while increasing omega-3 fatty acids (and related instruments) in cluster 2 shown effects on increased levels of glucose and inflammation indicators (Fig. 3A).

In the second step, we conducted multivariable MR analyses to estimate the direct effect of omega-3 fatty acids on T2DM conditioned on intermediate phenotypes in each cluster (Additional file 1: Table S5). For cluster 1, protection effects of omega-3 fatty acids on T2DM (OR: 0.59; 95%CI: 0.55–0.63; P = 4.64 × 10^–6^) were independent to HOMA-IR, but not FPG, FI, 2 h-PG, triglycerides, and LDL. For cluster 2, the direct effects of omega-3 fatty acids concentrations on T2DM conditioned on LDL, WBC, basophil percentage, or monocyte percentage were comparable between the univariable and multivariable MR estimates. It provided little evidence of a risk increasing effect of omega-3 fatty acids on T2DM conditioned on FPG, HOMA-B, triglycerides, or CRP. Multivariable MR controlling the effect of omega-3 fatty acids suggested that increasing HOMA-IR increased risk of T2DM (OR: 2.52; 95%CI 1.90–3.33; P = 6.52 × 10^–4^), while increasing HOMA-B decreased risk of T2DM (OR: 0.25; 95%CI 0.11–0.56; P = 0.008). The results were still significant after using the FDR method to correct the p-value. In outline, genetic variants increasing omega-3 fatty acid levels at least partly decrease T2DM risk via decreasing HOMA-IR in cluster 1, while increase T2DM risk via decreasing HOMA-B in cluster 2 (Fig. 3B).

## Discussion

Omega-3 supplementation is controversial for the prevention of cardiovascular metabolism disease, while T2DM is a heterogeneous disease with various pathogenic mechanisms. Here, we identified two distinct causal effects of omega-3 fatty acids on T2DM risk by using MR-Clust, highlighting different underlying biological/pleiotropic pathways in the association between omega-3 fatty acids and T2DM. Cluster 1 with 7 variants suggested that genetically-predicted higher circulating omega-3 fatty acids reduced FPG, FI, HOMA-IR and T2DM risk. On the contrary, cluster 2 with 10 instruments suggested that genetically-predicted higher circulating omega-3 fatty acid levels showed an effect on decreased HOMA-B, and increased FPG, inflammation and T2DM risk. Multivariable MR further suggested that the estimated effect of omega-3 fatty acids on T2DM was not independent to intermediate phenotypes such as FPG and triglycerides. Importantly, insulin resistance and beta cell dysfunction estimated using HOMA models might help discriminate the two distinct biological/pleiotropic pathways between omega-3 fatty acids related variants and T2DM. In summary, our study provided evidence to support the pleiotropic/heterogenous feature of omega-3 fatty acids instruments and distinct pathways linking omega-3 fatty acids with T2DM using genetics.

Previous observational studies revealed great heterogeneity for the association between omega-3 fatty acids and T2DM [9–14]. A meta-analysis of 83 randomized controlled trials suggested that omega-3 fatty acids had little effect on the prevention or treatment of T2DM and glucose metabolism [9]. A global consortium of large prospective studies indicated that circulating omega-3 fatty acids were associated with lower risk of T2DM [10]. On the contrary, it was reported that marine omega-3 fatty acids consumption was associated with higher T2DM risk in the Chinese population [37]. Such disparate findings from observational studies are likely due to residual confounding, reverse causality, and diverse study population. In the present study, we utilized the advanced feature of MR approach to control the influence of confounding and reverse causality when evaluating the effect of omega-3 fatty acids on T2DM risk.

However, MR has its own assumptions, which heterogeneity, including directional pleiotropy, is a key issue that need to be carefully accessed [38]. Previous studies support strong phenotypic and genetic correlation between fatty acids [21] and heterogeneity of the instruments of omega-3 fatty acids [20, 22, 23]. Indeed, shared enzymes, such as fatty acid desaturases encoded by *FADS1/2*, are involved in or even the rate-limiting enzymes for the metabolism of almost all polyunsaturated fatty acids including omega-3 and omega-6 fatty acids [39]. A recent study using conventional MR approaches suggested little evidence to support a causal effect of omega-3 fatty acids on T2DM in the general population [23]. Another study focused on the different components of omega-3 fatty acids, found that some omega-3 fatty acids subtypes were associated with lower risk of T2DM, while others were associated with higher risk or ineffectiveness [24]. In this study, we also observed weak evidence to support an overall causal effect of omega-3 fatty acids on T2DM, but found strong evidence to support heterogeneity of the MR estimate. We therefore systematically analyzed the heterogeneity/pleiotropy feature of omega-3 fatty acids instruments and its heterogenous effects on T2DM using MR-Clust and successfully divided the instruments of omega-3 fatty acids into two distinct clusters that estimated opposite directions of effects of omega-3 fatty acid levels on T2DM. After clustering the effects, the level of heterogeneity was notably attenuated. In addition, we showed that genetic variants belonging to each cluster were related to different intermediate phenotypes, illuminating the nature of pleiotropic effects of genetic variants influencing circulating omega-3 fatty acids. This suggests caution in the interpretation of MR studies of circulating omega-3 fatty acids given the possibility of ubiquitous pleiotropy effects highlighted by this and previous studies [21, 22, 40]. MR-Clust provides an innovative strategy, which can detect clusters of heterogenous effects of an exposure on an outcome [25]. We demonstrated the power of this method on dissecting heterogeneity using the relationship of omega-3 fatty acids and T2DM as an example.

MR-Clust also showed potentials to explore distinct mechanisms by which an exposure influences an outcome. In this study, we developed a new pipeline that integrating MR-Clust with two-step MR to identify distinct mechanisms linking omega-3 fatty acids variants with T2DM. For glycemic traits, cluster 1 instruments indicated an effect of increasing omega-3 fatty acids levels on lowering FPG, FI and HOMA-IR, which further reduced T2DM risk. But using cluster 2 instruments, increasing omega-3 fatty acids were estimated to have an effect on increased FPG levels and reduced HOMA-B levels, which further increased T2DM risk. Our results from the multivariable MR further suggested that HOMA-IR and HOMA-B were independently associated with T2DM even after controlling the omega-3 fatty acids effect. This implies that omega-3 fatty acids are likely to show a causal/pleiotropic effect of preserving insulin sensitivity in cluster 1, while exacerbated beta cell function in cluster 2. A recent meta-analysis of RCTs confirmed the supplementation with omega-3 had beneficial results on FPG and insulin resistance [41]. Previous mechanistic study suggested that omega-3 fatty acids caused GPR120-mediated anti-inflammatory and insulin sensitizing effects in vivo [15]. Therefore, given the discrepant effects between clusters, our findings reflect the pleiotropic nature of omega-3 fatty acids variants but may also reflect the distinct effects of omega-3 fatty acids on glucose homeostasis.

Another driving phenotype that could discriminate between the two clusters was serum triglycerides. Interestingly, omega-3 fatty acids variants protect against T2DM but predispose to increased triglycerides in cluster 1, while increased T2DM risk but moderately decreased triglycerides in cluster 2. We observed almost contradictory effects on lipid and glucose metabolism for omega-3 fatty acids in both two clusters. This paradoxical observation of glucose and lipid metabolism might can be partially explained by potential protection effect of triglycerides on the risk of T2DM. Findings from a previous MR study [42] suggested that triglycerides may show a causal effect on lowering risk of T2DM, although the effect was marginal. Apart from the influence of triglycerides, the *GCKR* variant, which was reported to be associated with decreased risk of T2DM but increased risk of dyslipidemia and fatty liver disease [43], may also play an important role to explain the pleiotropic pathways been identified. Detailed mechanisms on the association between omega-3 fatty acids, lipids and T2DM warrants to be explored in future studies.

For inflammation indicators, omega-3 fatty acids variants were associated with increased CRP and WBC in cluster 2. Previous studies suggested that omega-3 fatty acids could modulate inflammation by regulating mitochondrial function and endoplasmic reticulum stress [44]. Although we did not observe significant causal association between CRP and T2DM in the multivariable MR analysis, it did not preclude the possible that omega-3 fatty acids variants may affect T2DM risk through other inflammation indicators.

Although trials investigating the effect of omega-3 fatty acids interventions on T2DM suggested potential benefit of lipid metabolism [45, 46], inconsistent effects on glycemic control and T2DM risks were reported [9, 41]. Until now, there was insufficient evidence to establish the effect of omega-3 fatty acids on the risk of T2DM. Our study demonstrates the pleiotropic feature of omega-3 fatty acids variants and its complex effects on T2DM. Whether fish oil and/or omega-3 fatty acids supplementary can be used as interventional target for prevention and/or treatment of T2DM needs further validation that taking the pleiotropic effect into account. Omega-3 fatty acids using green nanomaterials to targeting cells and cell junctions may provide alternative future target therapeutics [47, 48]. As a main site for fatty acid β-oxidation, mitochondria play a central role in fatty acid metabolism. Understanding the molecular mechanisms that regulate mitochondrial function and omega-3 fatty acids may provide novel therapeutic targets for T2DM treatment [49]. Precise effect of omega-3 fatty acids on T2DM should be investigated in future genetic and clinical studies.

Our study has several strengths. We firstly use the method of MR-Clust which can identify variants that reflect heterogenous/pleiotropic feature of genetic instruments, and identify distinct causal mechanisms by integrating MR-Clust with two-step MR [25]. Based on this method, we identified two distinct causal effects of omega-3 fatty acids on T2DM risk for the first time, which highlight the need for careful interpretation of previous MR studies. Furthermore, we used the largest GWAS samples available for the omega-3 fatty acids, intermediate phenotypes and T2DM, which guarantees sufficient and powerful instrument selection.

Several limitations of this study should be acknowledged. First, the present MR analysis used GWAS data derived from populations of European ancestry. Hence, further multi-ancestry studies are needed to evaluate whether our findings can be generalized to individuals of other ancestries. Second, limited number of genetic instruments are available for HOMA-IR and HOMA-B, and weak instrument bias might exist in our multivariable MR analyses for HOMA-IR, which restricted our ability to identify the causal effect of the two glycemic traits on T2DM. Future efforts could be made to generate large-scale GWAS for HOMA-IR and HOMA-B. Third, the participants are partly overlap in the analysis of omega-3 fatty acids and T2DM, which may induce overfitting bias and lead to an estimate biased in the direction of the observational association. However, the overlap ratio was 33.6%, which is low. Therefore, the influence of sample overlap will be limited. Besides, after harmonization, only part of the omega-3 fatty acids variants (or LD proxies) can be found in the outcome data, therefore we were not able to use the full list of 52 instruments found in previous study [26]. Forth, we assume linear relationship between omega-3 fatty acids and intermediate phenotypes on T2DM in this study. Further non-linear Mendelian randomization analyses are needed to understand the potential non-linear effect of omega-3 fatty acids and intermediate phenotypes on T2DM. Another limitation of our study is that although MR-Clust provides a new angle to identify distinct causal effects, it is still difficult to fully distinguish the pleiotropic effects from distinct biological mechanisms linking the causal effect of omega-3 fatty acids on T2DM.

## Conclusions

We identified opposite causal effects of omega-3 fatty acid levels on T2DM risk estimated by two different cluster of genetic variants. One cluster of variants ameliorate insulin resistance and further decreased FPG, FI level and risk of T2DM, while the other one might relate to beta cell dysfunction further increased FPG level and T2DM risk. These findings highlight the heterogenous feature of omega-3 fatty acids instruments and its complex effects on T2DM, which need to be carefully considered in future genetic studies.

## Supplementary Information

**Additional file 1: Table S1.** Details of studies and datasets used for Two-sample Mendelian randomization analyses. **Table S2.** Characteristics of the selected genetic instruments for omega-3 fatty acids. **Table S4.** Clustered causal estimates of omega-3 fatty acids on potential intermediate risk factors. **Table S5.** Multivariable Mendelian randomization analyses estimating the direct effects of omega-3 fatty acids on T2DM, conditioning on intermediate risk factors. **Fig S1.** Scatter plot for the genetic liability of omega-3 fatty acids on type 2 diabetes. **Fig S2.** Radial plots of estimates between omega-3 fatty acids and triglycerides in Cluster 2.

**Additional file 2 Table S3.** Instruments used in the multivariable MR analysis.

## Data Availability

The GWAS summary statistics used in the current study are available from the IEU OpenGWAS database (https://gwas.mrcieu.ac.uk/) or the primary study website. More detailed information could be found in Additional file 1: Table S1.

## Abbreviations

CRP: C-reactive protein
FDR: False discovery rate
FPG: Fasting plasma glucose
FI: Fasting insulin
GWAS: Genome-wide association study
IVW: Inverse variance weighted
LD: Linkage disequilibrium
MR: Mendelian randomization
MR-PRESSO: MR Pleiotropy RESidual sum and outlier test
T2DM: Type 2 diabetes mellitus
WBC: White blood cell
2 h-PG: 2h-postload glucose
