# Supplementary material for: Evaluating the distinct pleiotropic effects of omega-3 fatty acids on type 2 diabetes mellitus: a mendelian randomization study
Source: J Transl Med. 2023 Jun 7;21:370. doi: 10.1186/s12967-023-04202-7 (PMC10249205; doi:10.1186/s12967-023-04202-7)

**Online Additional file materials**

1. **Additional file** 1: Table S1. Details of studies and datasets used for Mendelian randomization analyses.
2. **Additional file** 1: Table S2. Characteristics of the selected genetic instruments for omega-3 fatty acids.
3. **Additional file** 1: Table S3. Instruments used in the multivariable MR analysis (see **Additional file2:** Table S3.xlsx).
4. **Additional file** 1: Table S4. Clustered causal estimates of Omega-3 fatty acids on potential intermediate risk factors.
5. **Additional file** 1: Table S5. Multivariable Mendelian randomization analyses estimating the direct effects of omega-3 fatty acids on T2DM, conditioning on intermediate risk factors.
6. **Additional file** 1: Figure S1. Scatter plot for the genetic liability of omega-3 fatty acids on type 2 diabetes.
7. **Additional file** 1: Figure S2. Radial plots of estimates between omega-3 fatty acids and triglycerides in Cluster 2.

**Additional file 1: Table S1. Details of studies and datasets used for Mendelian randomization analyses.**

| **Phenotype** | **Ieugwas ID** | **Consortium or cohort study** | **Participants** | **Reference study description/ Website** |
| --- | --- | --- | --- | --- |
| *n*-3 PUFA | met-d-Omega_3 | UKB | 114,999 individuals of European ancestry | <https://gwas.mrcieu.ac.uk/datasets/met-d-Omega_3/>  doi: 10.1186/s12916-022-02399-w |
| Type 2 diabetes | ebi-a-GCST006867 | DIAGRAM, GERA and UKB data sets after quality controls | 655,666 individuals (62,892 T2DM cases and 596,424 controls) of European ancestry | doi: 10.1038/s41467-018-04951-w |
| Glycemic traits (FG, 2h-PG, FI and HbA1c) | / | MAGIC | European ancestry without diabetes | <http://magicinvestigators.org/downloads/>  doi: 10.1038/s41588-021-00852-9 |
| HOMA-IR, HOMA-B | HOMA-IR: ieu-b-118  HOMA-B: ieu-b-117 | MAGIC | European ancestry without diabetes, with fasting glucose <7 mmol/L | <http://magicinvestigators.org/downloads/>  doi:10.1038/ng.520 |
| Lipids (TG, HDL, LDL) | / | GLGC | 930,672 non-UKB European ancestry | http://csg.sph.umich.edu/willer/public/glgc-lipids2021  doi: 10.1038/s41586-021-04064-3 |
| CRP | ieu-b-35 | Meta-analysis of GWASs from CHARGE Inflammation Working Group | 204,402 European individuals from 88 studies | doi: 10.1161/CIRCGENETICS.108.829747  doi: 10.1016/j.ajhg.2018.09.009 |
| WBC traits | WBC count: ebi-a-GCST004610  Eo%: ebi-a-GCST004600  Mono%: ebi-a-GCST004609  Basop%: ebi-a-GCST004631  Lymph%: ebi-a-GCST004632  Neut%: ebi-a-GCST004633 | UKB, UK BiLEVE, INTERVAL | 173,480 European-ancestry participants | doi: 10.1016/j.cell.2016.10.042 |

DIAGRAM, DIAbetes Genetics Replication And Meta-analysis; GERA, Genetic Epidemiology Research on Adult Health and Aging. MAGIC, Meta-Analyses of Glucose and Insulin-related traits Consortium; GLGC, Global Lipids Genetics Consortium; CHARGE, the Cohorts for Heart and Aging Research in Genomic Epidemiology; UKB, UK Biobank. FPG, fasting plasma glucose; FI, fasting insulin; 2h-PG, 2h-postload glucose; TG, triglycerides; CRP, C-reactive protein; WBC, white blood cell. Baso%, Basophil percentage; Eo%, Eosinophil percentage; Lymph%, Lymphocyte percentage; Mono%, Monocyte percentage; Neut%, Neutrophil percentage.

**Additional file 1: Table S2. Characteristics of the selected genetic instruments for omega-3 fatty acids**

| **SNP** | **Nearby Gene(s)** | **Cluster** | **Probability** | **Effect Allele** | **Other Allele** | **EAF** | **Beta** | **SE** | **P** |
| --- | --- | --- | --- | --- | --- | --- | --- | --- | --- |
| rs1260326 | GCKR | 1 | 1.000 | C | T | 0.604 | -0.082 | 0.004 | 8.40×10^-88^ |
| rs58542926 | TM6SF2 | 1 | 0.993 | T | C | 0.074 | -0.172 | 0.008 | 1.40×10^-113^ |
| rs9987289 | LOC157273 | 1 | 0.988 | G | A | 0.909 | 0.057 | 0.007 | 3.20×10^-16^ |
| rs7924036 | JMJD1C | 1 | 0.804 | T | G | 0.504 | 0.023 | 0.004 | 5.50×10^-10^ |
| rs673335 | LOC105369393 | 1 | 0.702 | C | T | 0.160 | -0.067 | 0.006 | 1.10×10^-34^ |
| rs1672811 | MPV17L | 1 | 0.493 | C | T | 0.748 | 0.025 | 0.005 | 3.00×10^-08^ |
| rs6601924 | AKR1C4 | 1 | 0.448 | C | T | 0.846 | 0.035 | 0.006 | 8.50×10^-10^ |
| rs964184 | ZPR1 | 2 | 1.000 | C | G | 0.867 | -0.117 | 0.006 | 8.90×10^-87^ |
| rs174564 | FADS2 | 2 | 1.000 | G | A | 0.347 | -0.337 | 0.004 | 1.00×10^-200^ |
| rs72789541 | PDXDC1 | 2 | 0.999 | A | T | 0.296 | -0.081 | 0.004 | 5.60×10^-75^ |
| rs633695 | LIPC | 2 | 0.978 | G | A | 0.292 | 0.084 | 0.004 | 9.10×10^-80^ |
| rs1167998 | DOCK7 | 2 | 0.897 | A | C | 0.645 | 0.071 | 0.004 | 3.60×10^-66^ |
| rs1800978 | ABCA1 | 2 | 0.856 | G | C | 0.124 | -0.037 | 0.006 | 5.20×10^-09^ |
| rs117143374 | PSMG1 | 2 | 0.821 | C | T | 0.142 | -0.037 | 0.006 | 2.20×10^-10^ |
| rs35135293 | RPS16P2 | 2 | 0.695 | T | C | 0.517 | -0.021 | 0.004 | 3.90×10^-08^ |
| rs9304381 | LOC105372112 | 2 | 0.650 | T | C | 0.818 | 0.053 | 0.005 | 5.20×10^-24^ |
| rs4000713 | MIR148A; LOC105375199 | 2 | 0.567 | A | G | 0.295 | -0.029 | 0.004 | 1.00×10^-11^ |
| rs7819706 | LPL | 3 | 1.000 | G | A | 0.118 | -0.040 | 0.006 | 1.80×10^-10^ |
| rs7970695 | HNF1A | 3 | 0.991 | A | G | 0.621 | -0.025 | 0.004 | 1.20×10^-10^ |
| rs261290 | LIPC | 4 | 1.000 | C | T | 0.655 | -0.114 | 0.004 | 3.90×10^-161^ |
| rs62466318 | MLXIPL | 4 | 0.953 | T | C | 0.204 | -0.072 | 0.005 | 1.20×10^-45^ |
| rs10184054 | APOB | 4 | 0.617 | G | C | 0.224 | -0.036 | 0.005 | 5.60×10^-15^ |
| rs629301 | CELSR2 | 4 | 0.582 | T | G | 0.778 | 0.038 | 0.005 | 1.30×10^-14^ |
| rs10455872 | LPA | 4 | 0.525 | G | A | 0.079 | -0.063 | 0.008 | 2.80×10^-17^ |
| rs1132899 | APOC4-APOC2 | 4 | 0.475 | C | T | 0.510 | 0.027 | 0.004 | 8.60×10^-11^ |
| rs55891451 | CYP2C9 | 4 | 0.473 | C | A | 0.202 | 0.034 | 0.005 | 4.60×10^-12^ |
| rs6129624 | MAFB | 4 | 0.468 | A | G | 0.335 | -0.026 | 0.004 | 5.10×10^-10^ |
| rs11563251 | UGT1A1; UGT1A3-10 | 4 | 0.459 | T | C | 0.111 | 0.035 | 0.006 | 3.20×10^-08^ |
| rs11242109 | SLC22A4; MIR3936HG | 4 | 0.459 | T | G | 0.479 | 0.024 | 0.004 | 2.40×10^-09^ |
| rs6882345 | TIMD4 | 4 | 0.450 | A | G | 0.633 | 0.029 | 0.004 | 1.90×10^-13^ |

**Additional file 1: Table S4. Clustered causal estimates of Omega-3 fatty acids on potential intermediate risk factors.**

|  |  | **Cluster 1** | | |  |  | **Cluster 2** | | |  |
| --- | --- | --- | --- | --- | --- | --- | --- | --- | --- | --- |
| **Outcome** | **No. SNPs** | **Beta** | **SE** | **P value** | **FDR^†^** | **No. SNPs** | **Beta** | **SE** | **P value** | **FDR^†^** |
| FPG | 7 | **-0.18** | **0.07** | **0.006** | **0.021** | 10 | **0.04** | **0.007** | **3.49×10^-10^** | **2.09×10^-9^** |
| FI | 7 | **-0.16** | **0.06** | **0.007** | **0.021** | 10 | -0.01 | 0.008 | 0.216 | 0.259 |
| 2h-PG | 7 | **0.34** | **0.16** | **0.029** | **0.044** | 10 | 0.05 | 0.02 | 0.067 | 0.129 |
| HbA1c | 7 | -0.02 | 0.02 | 0.289 | 0.289 | 10 | 0.01 | 0.009 | 0.086 | 0.129 |
| HOMA-B | 7 | -0.04 | 0.02 | 0.131 | 0.157 | 5 | **-0.04** | **0.01** | **4.52×10^-05^** | **1.36×10^-4^** |
| HOMA-IR | 7 | **-0.13** | **0.05** | **0.018** | **0.036** | 5 | -0.007 | 0.01 | 0.544 | 0.544 |
| TG | 7 | **0.67** | **0.26** | **0.010** | **0.015** | 8^*^ | **-0.12** | **0.06** | **0.034** | 0.051 |
| HDL | 7 | 0.11 | 0.17 | 0.514 | 0.514 | 10 | 0.13 | 0.12 | 0.275 | 0.275 |
| LDL | 7 | **0.40** | **0.10** | **0.0001** | **0.0003** | 10 | **0.15** | **0.04** | **0.0003** | **0.0009** |
| CRP | 7 | 0.31 | 0.20 | 0.130 | 0.320 | 8 | **0.05** | **0.01** | **1.95×10^-04^** | **6.83×10^-4^** |
| WBC | 7 | 0.11 | 0.14 | 0.426 | 0.668 | 10 | **0.05** | **0.02** | **0.002** | **4.67×10^-3^** |
| Baso% | 7 | -0.01 | 0.07 | 0.905 | 0.929 | 10 | **-0.03** | **0.01** | **0.014** | **0.025** |
| Eo% | 7 | -0.09 | 0.05 | 0.095 | 0.320 | 10 | 0.03 | 0.03 | 0.418 | 0.456 |
| Lymph% | 7 | 0.06 | 0.09 | 0.477 | 0.668 | 10 | -0.01 | 0.02 | 0.456 | 0.456 |
| Mono% | 7 | -0.19 | 0.13 | 0.137 | 0.320 | 10 | **-0.08** | **0.01** | **3.33×10^-14^** | **2.33×10^-13^** |
| Neut% | 7 | 0.01 | 0.11 | 0.929 | 0.929 | 10 | 0.03 | 0.02 | 0.169 | 0.237 |

FPG, fasting plasma glucose; FI, fasting insulin; 2h-PG, 2h-postload glucose; TG, triglycerides; CRP, C-reactive protein; WBC, white blood cell. Baso%, Basophil percentage; Eo%, Eosinophil percentage; Lymph%, Lymphocyte percentage; Mono%, Monocyte percentage; Neut%, Neutrophil percentage. FDR: p-value adjusted using Benjamini-Hochberg False Discovery Rate. ^*^rs964184 and rs1167998 were removed for over-dispersion based on Radial MR analysis.

**Additional file 1: Table S5. Multivariable Mendelian randomization analyses estimating the direct effects of omega-3 fatty acids on type 2 diabetes, conditioning on intermediate risk factors.**

|  |  | **Number of SNP instruments** | **Number of SNPs in analysis** | **Conditional**  **F statistic** | **IVW odds ratio (95% CI)** | **P value** | **FDR** |
| --- | --- | --- | --- | --- | --- | --- | --- |
| **Cluster 1** | Omega-3 | 7 | 59 | 121.21 | 0.97 (0.70-1.33) | 0.83 | / |
|  | FPG | 65 | 59 | 118.11 | 1.48 (0.66-3.32) | 0.35 | 0.63 |
|  | Omega-3 | 7 | 36 | 33.97 | 0.53 (0.22-1.25) | 0.16 | / |
|  | FI | 38 | 36 | 46.92 | 0.85 (0.17-4.13) | 0.84 | 0.84 |
|  | Omega-3 | 7 | 16 | 46.48 | 0.44 (0.12-1.58) | 0.23 | / |
|  | 2h-PG | 14 | 16 | 27.79 | 1.49 (0.52-4.24) | 0.47 | 0.63 |
|  | Omega-3 | 7 | 8 | 21.11 | **0.59 (0.55-0.63)** | 4.64**×**10^-6^ | / |
|  | HOMA-IR^*^ | 3 | 8 | 6.71 | **2.52 (1.90-3.33)** | 6.52**×**10^-4^ | 0.003 |
|  | Omega-3 | 7 | 275 | 35.80 | 0.98 (0.86-1.11) | 0.74 | / |
|  | TG | 377 | 275 | 77.87 | 1.04 (0.88-1.23) | 0.63 | 0.95 |
|  | Omega-3 | 7 | 204 | 37.53 | 0.96 (0.89-1.05) | 0.39 | / |
|  | LDL | 316 | 204 | 65.98 | 1.00 (0.89-1.13) | 0.95 | 0.95 |
| **Cluster 2** | Omega-3 | 10 | 63 | 228.60 | 1.07 (0.86-1.34) | 0.54 | / |
|  | FPG | 65 | 63 | 113.34 | 1.43 (0.66-3.32) | 0.36 | 0.36 |
|  | Omega-3 | 10 | 12 | 319.93 | 1.05 (0.91-1.22) | 0.53 | / |
|  | HOMA-B | 4 | 12 | 24.84 | **0.25 (0.11-0.56)** | 0.008 | 0.016 |
|  | Omega-3 | 10 | 278 | 61.53 | 1.08 (0.98-1.19) | 0.11 | / |
|  | TG | 377 | 278 | 134.85 | 1.11 (0.96-1.27) | 0.16 | 0.32 |
|  | Omega-3 | 10 | 210 | 63.79 | **1.08 (1.01-1.15)** | 0.02 | / |
|  | LDL | 316 | 210 | 81.16 | 1.01 (0.91-1.14) | 0.80 | 0.80 |
|  | Omega-3 | 10 | 58 | 145.35 | 1.03 (0.89-1.18) | 0.72 | / |
|  | CRP | 57 | 58 | 140.39 | 1.00 (0.85-1.16) | 0.96 | 0.96 |
|  | Omega-3 | 10 | 138 | 102.25 | **1.07 (1.01-1.12)** | 0.02 | / |
|  | WBC | 179 | 138 | 64.13 | 0.96 (0.88-1.04) | 0.27 | 0.72 |
|  | Omega-3 | 10 | 48 | 166.79 | **1.09 (1.03-1.16)** | 0.008 | / |
|  | Baso% | 65 | 48 | 66.19 | 1.04 (0.92-1.16) | 0.54 | 0.72 |
|  | Omega-3 | 10 | 129 | 107.90 | **1.06 (1.00-1.12)** | 0.04 | / |
|  | Mono% | 194 | 129 | 97.11 | 1.02 (0.96-1.09) | 0.50 | 0.72 |

FPG, fasting plasma glucose; FI, fasting insulin; 2h-PG, 2h-postload glucose; TG, triglycerides; CRP, C-reactive protein; WBC, white blood cell; Baso%, Basophil percentage; Mono%, Monocyte percentage. FDR: p-value adjusted using Benjamini-Hochberg False Discovery Rate. ^*^Instruments selection used a more lenient threshold of P<10^-6^.

**Additional file 1: Figure S1.** **Scatter plot for the genetic liability of omega-3 fatty acids on type 2 diabetes.**


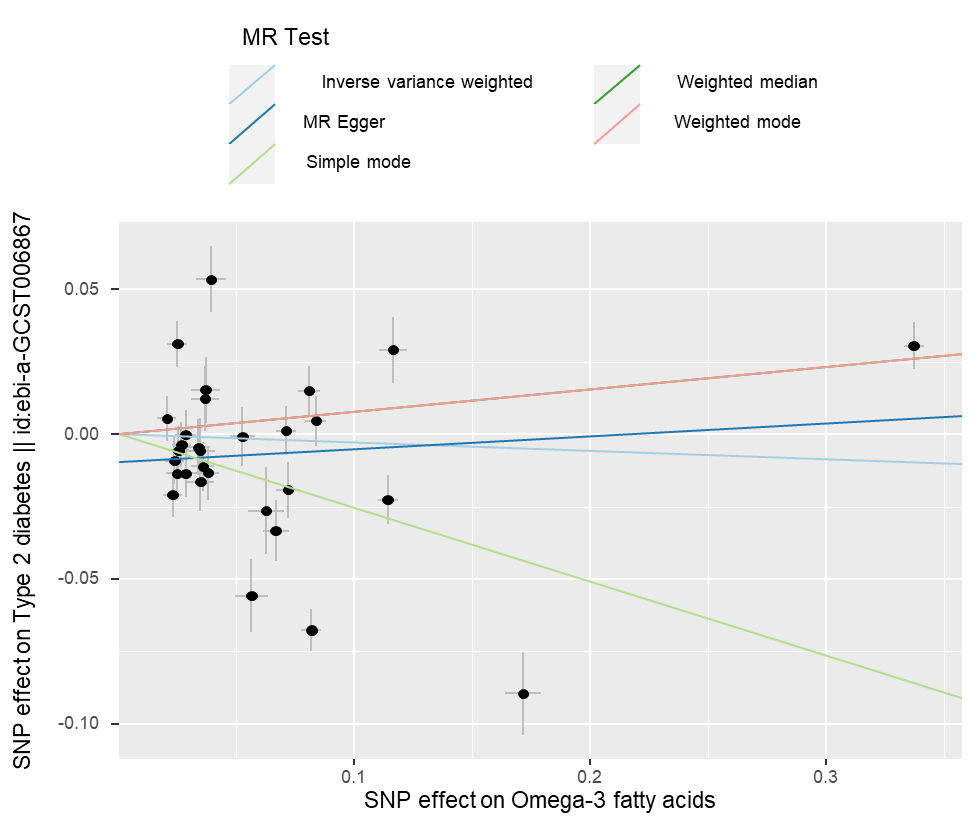


**Additional file 1: Figure S2. Radial plots of estimates between omega-3 fatty acids and triglycerides in Cluster 2**.


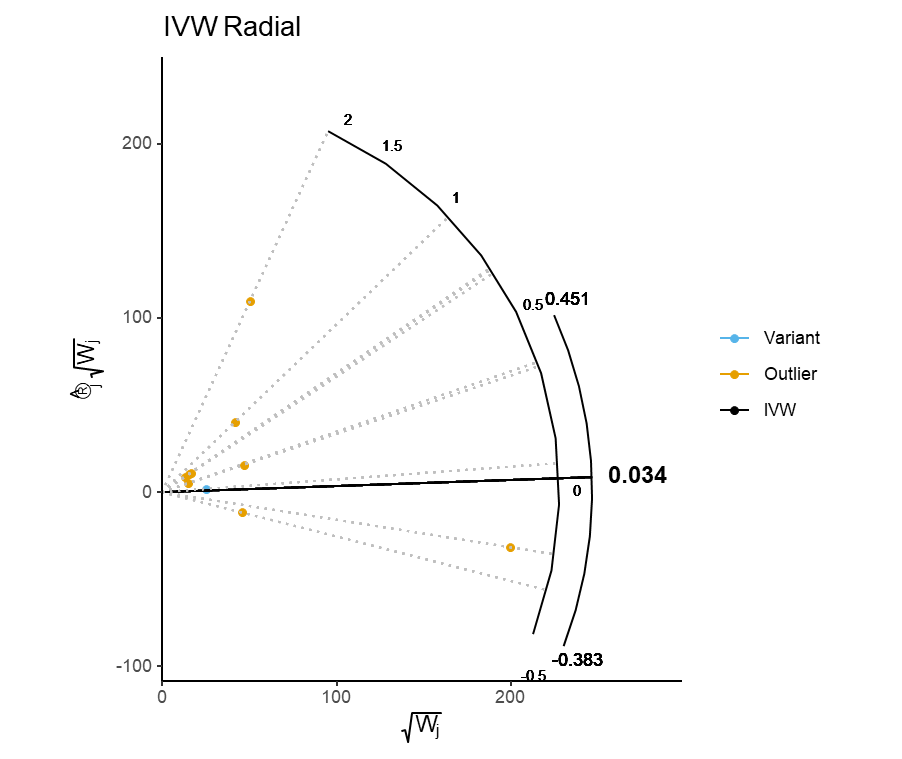

Supplement: Supplementary file 1 — Additional file 1: Table S1. Details of studies and datasets used for Two-sample Mendelian randomization analyses. Table S2. Characteristics of the selected genetic instruments for omega-3 fatty acids. Table S4. Clustered causal estimates of omega-3 fatty acids on potential intermediate risk factors. Table S5. Multivariable Mendelian randomization analyses estimating the direct effects of omega-3 fatty acids on T2DM, conditioning on intermediate risk factors. Fig S1. Scatter plot for the genetic liability of omega-3 fatty acids on type 2 diabetes. Fig S2. Radial plots of estimates between omega-3 fatty acids and triglycerides in Cluster 2. [file 12967_2023_4202_MOESM1_ESM.docx]
